# Safety and aesthetic outcomes of SERASYNTH^Ⓡ^ MESH BR for direct-to-implant breast reconstruction: A retrospective single center analysis of 32 consecutive cases

**DOI:** 10.1016/j.jpra.2023.08.001

**Published:** 2023-08-16

**Authors:** Julia Gruber, Paul Schlagnitweit, Georgios Koulaxouzidis

**Affiliations:** a Department of Plastic, Aesthetic and Reconstructive Surgery, Sisters of Mercy Hospital Linz, Austria; b Department of Plastic, Aesthetic and Reconstructive Surgery, Hospital Wels-Grieskirchen, Austria

**Keywords:** Prophylactic mastectomy, Direct-to-implant breast reconstruction, Serasynth mesh, Mesh resorption time, Seroma

## Abstract

**Background:**

Bilateral mastectomy for both therapeutic and prophylactic reasons is becoming increasingly important. To achieve good results after mastectomy, synthetic meshes are often used as an alternative to acellular dermal matrices (ADMs). The aim of this study is to analyze the results of subcutaneous mastectomies and direct-to-implant breast reconstruction using SERASYNTH^Ⓡ^ MESH BR.

**Methods:**

In this work, data from mastectomies (*n* = 32) in 22 patients without prior radiation after breast reconstruction with SERASYNTH^Ⓡ^ MESH BR from a single center were retrospectively analyzed with 1 year follow-up. Complications were categorized as serious (need for revision surgery) and minor events. Statistical analysis was performed using the *t*-test in SPSS. Data were compared with the existing literature.

**Results:**

Major complications occurred in 15.6% (*n* = 5). Two out of five revisions were due to hematoma. In three cases, a seroma followed by other complications (e.g., infections, necrosis) necessitated revision. Minor complications occurred in 12.5% of cases. Due to the safety aspect, implants were replaced in each revision. There was no significant difference in complication rates between prophylactic and therapeutic mastectomies (*p* = 0.3815, SE = 0.171). There was no statistically significant difference in esthetic outcomes (*p* = 0.3846).

**Conclusion:**

The application of the absorbable polymer poly-p-dioxanone SERASYNTH^Ⓡ^ MESH BR has complication rates comparable to those reported in the existing literature. Careful patient selection is paramount in order to limit the complication rate. SERASYNTH^Ⓡ^ MESH BR can be considered a safe tool to achieve esthetic results in combination with direct-to-implant breast reconstruction.

## Introduction

As breast cancer is the most common cancer in women, with an incidence rate of 13.3%,^1^ mastectomy and direct-to-implant (DTI) breast reconstruction are becoming increasingly important. It is the most common method of breast reconstruction. More than 80% of women choose this method over reconstruction with autologous tissue.^2^

Bilateral mastectomies accounted for 4.4% of the total mastectomies in 1998, which increased to 22.4% by 2008. Studies suggest that the increase in bilateral mastectomies is not due to changes in the incidence of bilateral breast cancer but rather to the growing use of contralateral prophylactic mastectomy.^3^ Implant-based breast reconstruction has many advantages, although a supporting measure such as an acellular dermal matrix (ADM) or synthetic mesh is mostly required. The most important factor is that the patient's overall morbidity is lower because implant-based breast reconstruction is less invasive, the operative time is shorter, and there are fewer complications than with autologous reconstruction.^4^, ^5^, ^6^, ^7^ To achieve good results after a mastectomy, the implant must be well covered, especially if the skin envelope is thin. In addition, the position of the implant must be supported. For this reason, there are many techniques to support the skin, such as ADMs, which are the most commonly used worldwide, and synthetic meshes that are increasingly used nowadays.^8^ Better control of the inframammary fold, a more natural contour of the lower pole, and prevention of lateral implant migration can be achieved with a matrix or mesh for implant-based breast reconstruction.^9^, ^10^, ^11^

Synthetic meshes are gaining popularity due to their safety, efficacy, and low rate of capsular contracture.^12^, ^13^, ^14^ Breast reconstruction with synthetic mesh was first published in 1997 by Rietjens et al.^15^ Nowadays, a lot of approved meshes are available in the market^16^, ^17^, ^18^, ^19^, ^20^, ^21^, ^22^, ^23^ (excerpt Table 1). Compared to ADMs, there are fewer data on PubMed, and studies are often underpowered.^18^, ^19^, ^20^ Because of good clinical experiences, we principally used synthetic meshes, particularly the SERASYNTH ^Ⓡ^ MESH BR (Serag-Wiessner, Germany).^24^

**Table 1:** Different meshes used most frequently.

Meshes	Description	(Non-) absorbable/partially absorbable
TiLOOP^Ⓡ^Bra (pfm medical, Cologne, Germany)^21^	Titanium-coated polypropylene mesh, macroporous, light, monofil	Nonabsorbable
SERASYNTH^Ⓡ^ MESH (SERAG-WIESSNER, Germany)^22^	Polydioxanone, monofil, macroporous, light,	Totally absorbable in 30 weeks
SERAGYN^Ⓡ^ BR PA (SERAG-WIESSNER, Germany)^22^	Polypropylen/polyglycolsäure-caprolapton (PGACL), monofil, macroporous, light	Partially absorbable after 120 days (PGACL absorbs, PP remains)
VICRYL^Ⓡ^ MESH^23^	Polyglactin mesh	Totally absorbable after 90–180 days

## Materials and methods

### Patients

In this work, we evaluated data from subcutaneous mastectomies after breast reconstruction with implants and SERASYNTH^Ⓡ^ MESH BR from a single center. We collected data from 35 breast reconstructions performed on 25 patients. The exclusion criterion was previous radiotherapy, reducing the patient population to 32 breast reconstructions in 22 patients. All reconstructions were performed for oncological reasons - both prophylactic and curative. In case of a bilateral procedure, each side was counted separately. In one patient, only data on prophylactic mastectomy were available due to a two-stage procedure. All reconstructions were performed with implants (*n* = 31) or expanders (*n* = 1), subpectorally, and with SERASYNTH^Ⓡ^ MESH BR fixation. Postoperative outcomes were evaluated by three observers who rated the esthetic outcome on a numeric scale of one to ten, with ten representing the best outcome. The observers were three randomly selected plastic surgeons from our team who were not associated with the surgery.

### Aims

The endpoint of this study was serious or minor events. Serious events were defined as any complication that required revision surgery, with or without implant failure. Other retrospective points of interest were less serious events that did not require revision surgery. Hematomas, seromas, minor infections (without need for intravenous antibiotics?), and wound dehiscence were examples for minor events. Esthetic outcomes were compared between the prophylactic and therapeutic groups.

### Statistical evaluation

To evaluate the efficacy of SERASYNTH^Ⓡ^ MESH BR in breast reconstruction, we compared our data with those already published on PubMed. Special attention was paid to TiLOOP^Ⓡ^ Bra and SERAGYN^Ⓡ^ BR meshes.

A statistical comparison between prophylactic and therapeutic mastectomies was performed using *t*-test in SPSS^Ⓡ^, including for esthetic outcomes. Quantitative meta-analysis was not possible due to the heterogeneity of study designs and underreporting.

### Surgical techniques

Surgeries were performed according to current guidelines. When therapeutic skin-sparing mastectomy or nipple-sparing mastectomy was indicated, the surgery was performed by breast surgeons at the Breast Health Center. The prophylactic contralateral side was operated by an experienced plastic surgeon from our department. In terms of oncologic safety, special attention was paid to tissue-sparing mastectomy to avoid postoperative necrosis and wound infection. Prior to this, patients were informed about the options for breast reconstruction with implants or autologous tissue. A procedure was then recommended and evaluated considering various factors such as patient preference, body habitus, comorbidities, and previous abdominal surgery. Implants were placed submuscularly with a radial smart vertical or inframammary incision. The mesh was sutured to the inferior muscle edge with Vicryl 2-0 and either fixed in the submammary crease or wrapped under the implant.

Antibiotic therapy was administered intravenously in ten cases as single therapy when patients were at a very low risk of infection or when antibiotic therapy was not required based on clinical parameters. On average, intravenous antibiotic therapy was continued for 7 days (range = 1–16). Drains were used in all cases and were removed until there was less than 30 ml/24 h of wound fluid in them. The average length of stay was 8 days (range = 3–13).

#### SERASYNTH^Ⓡ^ MESH BR

SERASYNTH^Ⓡ^ MESH BR (SERAG-WIESSNER GmbH & Co. KG, Naila/Germany) is a fully absorbable, sterile surgical mesh made of synthetically produced, monofilament basic threads. It consists of polymeric poly-p-dioxanone, the thread material corresponds to the SERASYNTH^Ⓡ^ suture material. SERASYNTH^Ⓡ^ MESH BR is degraded in the tissue by hydrolysis and subsequently metabolized in the body. This resorption initially manifests itself in a decrease in mesh strength, which is later accompanied by a loss of mass. The tensile strength gradually decreases over time, and the mesh is completely resorbed after about 30 weeks.

## Results

Thirty-two DTI breast reconstructions with SERASYNTH^Ⓡ^ MESH BR were performed in 22 patients. Three procedures in three patients were excluded due to previous radiotherapy.

Patient characteristics are summarized in Table 2. Twenty-one were therapeutic (65.6%) and 11 were prophylactic (34.4%) mastectomies in patients at high risk for breast cancer (e.g., BRCA ½ mutation, positive family history).

**Table 2:** Patient's characteristics.

Procedures	*N* = 32
*Type of mastectomy*	
Therapeutic	21 (65.6%)
Prophylactic	11 (34.4%)
*Incision*	
Inframammary fold	14 (43.8%)
Radial	10 (31.3%)
Vertical	5 (15.6%)
Wise pattern	3 (9.3%)
*Surgery*	
Bilateral	21 (65.6%)
Unilateral	11 (34.4%)
*Resection weight, g (range)*	271.6 (101.0–741.0)*
*Age, y (range)*	44.3 (25.0–67.0)
*Smoking*	
Active or former smoker	4 (12.5%)
Never smokers	28 (87.5%)
*BMI, kg/m^2^ (range)*	22.4 (18.4–29.7)
*Comorbidities*	
Diabetes	1 (3.1%)
APC Resistance	1 (3.1%)
Autoimmune disease	1 (3.1%)
Anticoagulation	1 (3.1%)
*ASA classification*	
*ASA I*	20 (62.5%)
*ASA II*	12 (37.5%)
*ASA III-VI*	0

⁎ Data for six patients are missing.

Fourteen mastectomies (43.8%) were performed with an incision in the inframammary fold, ten (31.3%) with a radial incision, five (15.6%) with a vertical incision, and three (9.3%) with the wise pattern technique. Unilateral surgery was performed in 11 breast reconstructions (34.4%), and bilateral surgery was performed in 21 procedures (65.6%). The mean resection weight was 271.6 g (range = 101.0–741.0). The follow-up period was 1 year. The median age was 44.3 years (range = 25–67). Median body mass index (BMI) was 22.4 +/− 3.2 (range = 18.4–29.7). Four people were active or former smokers (12.5%).

A total of nine complications occurred in nine patients (Table 3). Minor complications occurred in 12.5% of cases. The infection rate was 3.1% and the hematoma rate was 9.3%. The necrosis and seroma rates were 3.1% each. There were five revision surgeries (15.6%). Two of the five revisions were due to a hematoma. There were three cases, in which several of the causes listed in Table 3, such as hematoma and skin necrosis due to skin tension and an accompanying infection, led to revision surgery, a rate of 9.3%. Two of the three cases had immediate postoperative complications. One was because of an infected seroma a few weeks postoperatively. Due to the safety aspect, implants were replaced at each revision. There was no significant difference in complication rate between prophylactic and therapeutic mastectomies (*p* = 0.3815; SE = 0.171). Esthetic outcomes were evaluated by three independent observers, who rated the results on a numerical scale of one to ten, with ten representing the best outcome. The mean value for therapeutic mastectomy was 7.5, for prophylactic mastectomy was 8.1. There was no significant difference between the two groups (*p* = 0.3846) (Table 4). There was no revision surgery due to esthetic results. Late complications such as capsular contracture or implant rupture were not observed, as the follow-up period was only 1 year.^25^

**Table 3:** Complications.

Complications	No. (%)
**Overall**	**9 (28.1%)**
Major complication	5 (15.6%)
Minor complications	4 (12.5%)
**In detail**	
Hematoma	3 (9.3%)
Infection	1 (3.1%)
Seroma	1 (3.1%)
Necrosis	1 (3.1%)
Multiple	3 (9.3%)

**Table 4:** Esthetic outcomes in VAS.

	Therapeutic	Prophylactic
Mean	7.5	8.1
Variance	2.6	3.7
*P* = 0.38		

Table 4 shows the esthetic scores of three observers on a VAS of 1 to 10, 10 being the best score.

VAS, visual analog scale.

## Discussion

With the introduction of DTI breast reconstruction with mesh support, improvements in surgical techniques and consequently esthetic outcomes could be achieved. Currently, there are no data that allow a direct comparison of different meshes. Due to the small number of participants, studies are often underpowered. The advantages of each mesh are only briefly discussed.^26^ Our study shows acceptable complication rates that are comparable to the existing literature. Complications are not necessarily due to the meshes, such as the occurrence of hematomas. Table 5 shows a comparison between different synthetic meshes. The high complication rate in our study might be due to the small number of patients. However, with an overall complication rate of 28.1%, our results are comparable to the TiLOOP^Ⓡ^ meshes in a study by Dieterich et al. (29.0%). The rate of serious complications was slightly higher in our study (15.6% vs. 13.4%) when postoperative hematomas after revision surgery were not excluded.^12^ When hematomas only indirectly related to the mesh are excluded, the major complication rate decreases to 9.3%. In our collective, there were slightly more serious complications and, conversely, a lower number of minor complications (12.5% vs. 15.6%). Considering the small sample sizes, these variations are to be expected. Different treatment approaches and when to perform revision must also be considered.^12^

**Table 5:** Comparison of different studies regarding complication rate.

	Major complications	Minor complications	Overall
Our study (SERASYNTH^Ⓡ^ MESH BR)	15.6	12.5	28.1
Dieterich et al. (TiLOOP^Ⓡ^)	13.4	15.6	29.0
Benini et al. (TiLOOP^Ⓡ^)	12.0	9.0	35.3
Quah et al. (TiLOOP^Ⓡ^)	7.8	2.2	14.0
Eichler et al. (SERAGYN^Ⓡ^)	3.9	18	21.8
Haynes et al. (VICRYL^Ⓡ^ Mesh)^23^	8.7	6.5	15.2

In a study by Bernini et al. 12 of 34 cases of breast reconstruction with TiLOOP^Ⓡ^ Bra resulted in implant exchange during a follow-up period of 26 months.^24^ Quah et al. described a major complication rate for the nonabsorbable TiLOOP^Ⓡ^ meshes of 7.8%.^21^

Overall complication rate for the partially absorbable SERAGYN^Ⓡ^ BR meshes was 21.8%. Serious complications occurred in 3.9% within the first 3 months, and the minor complication rate was 18%. The most common minor complication was a seroma in 9.4% of cases.^26^

Seroma is one of the most common postoperative complications of implant-based breast reconstruction with mesh fixation, with incidence rates ranging from 3% to 90%.^27^, ^28^, ^29^, ^30^ Mesh implantation is associated with a higher rate of seroma formation. Previous studies have shown that the use of synthetic mesh instead of ADMs, which have been commonly used in breast reconstruction, reduces the risk of seroma formation.^31^ The development of seroma increases the risk of subsequent complications such as infections, necrosis, implant rotation after implant loss.^32^ With an incidence rate of 12.5% for all seroma formations in our study, the results are comparable to the known literature. If the mesh is fully and more rapidly absorbed, seroma formation could also be lower. Further studies are needed to investigate the difference between fully absorbable and partially or nonabsorbable meshes.^12^^,^^24^

Grous et al. compared data on immediate prepectoral breast reconstruction after mastectomy with implants and the use of SERASYNTH^Ⓡ^ MESH BR and SERAGYN^Ⓡ^ BR mesh. In the SERASYNTH^Ⓡ^ MESH BR group and the SERAGYN^Ⓡ^ mesh group, reoperation was required in 5.1% and 5% of patients.^33^ Further studies would be interesting to analyze whether there are significant differences between meshes with different resorption times.

Overall, it was shown that there is little data in the existing literature comparing the individual meshes. A little light is shed on the differences between the meshes. Given the comparable complication rates, economic considerations can also be taken into account. Synthetic meshes are becoming increasingly popular, in part because of their lower cost. Studies show a low complication rate, excellent cosmetic outcome, and significant cost savings compared to the use of ADM.^16^^,^^17^^,^^34^, ^35^, ^36^

There was no statistically significant difference in complication rates between prophylactic and therapeutic mastectomy and DTI breast reconstruction with mesh insertion. Studies on this topic are rare. It would be of great importance to determine whether the skin layer in therapeutic mastectomy (which is often operated more traumatically due to the tumor) has an influence on the outcome. The thicker skin envelope is often noticeable during reconstruction because a smaller implant must be used. This is our own experience and not that described in the literature. Also, it would be interesting to see if there is a difference in resection weight and outcome between the two groups. In our study, the trend is toward prophylactic mastectomy, although there is no statistically significant difference. This could be due to the small patient population. The risk of developing breast cancer in women with a BRCA ½ mutation is between 60% and 80%. Prophylactic mastectomy can reduce the risk by up to 95%.^37^, ^38^, ^39^ If the complication rate of contralateral prophylactic mastectomy were lower than that of therapeutic mastectomies for bilateral mastectomy with DTI breast reconstruction, the indication could be more generous to reduce the long-term risk of contralateral breast cancer recurrence. Further studies are needed to clarify this issue.

To make the group more homogeneous, we excluded patients who had previously received radiotherapy or in whom radiotherapy was unclear. Adjuvant chemotherapy or radiotherapy is associated with an increased risk of postoperative complications, particularly rates of capsular contracture, infection, and implant loss.^40^, ^41^, ^42^ Careful planning of mastectomy and DTI breast reconstruction is important to reduce the complication rate. If radiotherapy is planned or cannot be ruled out postoperatively when planning the procedure, a two-stage procedure, possibly with autologous tissue, should be considered.^43^

Not only should the complication rate be analyzed but also the esthetic outcome, as it is as important as postoperative complications for patients to improve psychosocial function and body image.^44^ For this evaluation, we had three observers rate esthetic results using a visual analog scale, with a score from one to ten, with ten being the best score. To objectify the results, it would be best to include the patient's opinion with a validated quality of life questionnaire, such as Breast-Q, to evaluate the impact of the results also from the patient's perspective.

## Conclusions

The absorbable SERASYNTH^Ⓡ^ MESH BR made of poly-p-dioxanone, has complication rates comparable to those reported in the existing literature. To limit the complication rate, careful patient selection is of utmost importance. SERASYNTH^Ⓡ^ MESH BR can be considered a safe means of achieving esthetic results in combination with DTI breast reconstruction. There is a lack of studies comparing DTI breast reconstruction after prophylactic or therapeutic mastectomy, which would be of great interest to facilitate the indication for contralateral prophylactic mastectomy and DTI breast reconstruction. Because of the lower complication rate of synthetic meshes, which is comparable to ADMs, synthetic meshes are a safe tool for breast reconstruction, and the relatively lower cost relieves the burden on the healthcare system.
